# Inactivated ostreid herpesvirus-1 induces an innate immune response in the Pacific oyster, *Crassostrea gigas*, hemocytes

**DOI:** 10.3389/fimmu.2023.1161145

**Published:** 2023-04-28

**Authors:** Lizenn Delisle, Anne Rolton, Julien Vignier

**Affiliations:** ^1^ Biosecurity Group, Cawthron Institute, Nelson, New Zealand; ^2^ Aquaculture Group, Cawthron Institute, Nelson, New Zealand

**Keywords:** immune priming, Ostreid herpesvirus-1, pseudo-vaccination, innate immune memory, flow cytometry, reactive oxygen species, droplet digital PCR

## Abstract

Infectious diseases are a major constraint to the expansion of shellfish production worldwide. Pacific oyster mortality syndrome (POMS), a polymicrobial disease triggered by the Ostreid herpesvirus-1 (OsHV-1), has devastated the global Pacific oyster (*Crassostrea gigas*) aquaculture industry. Recent ground-breaking research revealed that *C. gigas* possess an immune memory, capable of adaption, which improves the immune response upon a second exposure to a pathogen. This paradigm shift opens the door for developing ‘vaccines’ to improve shellfish survival during disease outbreaks. In the present study, we developed an *in-vitro* assay using hemocytes – the main effectors of the *C. gigas* immune system – collected from juvenile oysters susceptible to OsHV-1. The potency of multiple antigen preparations (e.g., chemically and physically inactivated OsHV-1, viral DNA, and protein extracts) to stimulate an immune response in hemocytes was evaluated using flow cytometry and droplet digital PCR to measure immune-related subcellular functions and gene expression, respectively. The immune response to the different antigens was benchmarked against that of hemocytes treated with Poly (I:C). We identified 10 antigen preparations capable of inducing immune stimulation in hemocytes (ROS production and positively expressed immune- related genes) after 1 h of exposure, without causing cytotoxicity. These findings are significant, as they evidence the potential for priming the innate immunity of oysters using viral antigens, which may enable cost-effective therapeutic treatment to mitigate OsHV-1/POMS. Further testing of these antigen preparations using an *in-vivo* infection model is essential to validate promising candidate pseudo-vaccines.

## 1 Introduction

The occurrence of mass mortality events and the emergence of infectious diseases affecting marine organisms have increased dramatically in recent years, exacerbated by a changing environment (1–3). These outbreaks can have disastrous consequences on biodiversity and cause rapid population declines, particularly in cultured livestock (4, 5). Diseases are the major limiting factor for the expansion of the aquaculture industry, with losses attributed to infectious microbial diseases alone exceeding US$ 6 billion per annum (6). One striking example is Pacific oyster mortality syndrome (POMS), which is associated with the detection of the Ostreid Herpes virus-1 (OsHV-1) and its variants. Over the last 15 years, OsHV-1 has decimated Pacific oysters, *Crassostrea gigas*, worldwide (7; see for review 8–10). The virus induces an immune-compromised state in infected oysters, which evolves toward subsequent bacteremia by opportunistic bacterial pathogens, leading to mortality rates of up to 100% in juveniles (11). Recorded for the first time in France in 2008, OsHV-1 µvar rapidly spread along the European coastline (9, 10, 12), and closely related variants of the virus were further detected during mortality events in Australia (13), New Zealand (14), Korea (15), and more recently in California (16). The inability to contain the rapid spread of the virus combined with an absence of therapeutic treatments resulted in huge losses of aquaculture stocks. Selective breeding to improve resistance to POMS (or OsHV-1) has shown potential as a prevention strategy, with moderate to high heritability for survival achieved during OsHV-1 infection (17–20). Implementation of a breeding program and access to selectively bred stocks can, however, be economically challenging for many end-users, prompting the need for new, accessible, and complementary mitigation strategies to reduce the impact of diseases.

For example, vaccination and immune priming have proven to be an effective preventative measure for many major diseases affecting livestock, including fish, and more recently invertebrates, such as crustaceans (21–25).

Invertebrates lack a conventional adaptive immune system (i.e., lymphocytes or antibodies) and instead rely on innate immunity to prevent the infection of invading pathogens (26, 27). Numerous studies have reported that invertebrates also possess diverse forms of immune ‘memory’ in which a potentiated immune response (resulting in a reduction of host susceptibility to the infection) has been recorded following a secondary exposure to a pathogen (28–31). For instance, in the scallop *Chlamys farreri*, a first short exposure to the pathogen *Vibrio anguillarum* increased phagocytosis, acid phosphatase activity, and survival following a second encounter to the pathogen (32). Pacific oysters stimulated primarily by heat-killed *Vibrio splendidus* also displayed stronger immune responses at cellular and molecular levels when they were subjected to a secondary challenge with the live bacteria (33). (34, 35) showed that injection of Polyinosinic: polycytidylic acid or Poly (I:C), a synthetic analog of double stranded RNA with immunostimulant properties, induced a long-lasting antiviral response in Pacific oysters, protecting them against subsequent OsHV-1 infection in natura. More recently, Fallet et al. (36) showed that early life exposure of *C. gigas* to ‘microorganisms’ provided inter-generational protection against recurring OsHV-1 infections, indicating a potential trained immunity *via* epigenetic modifications. In bivalves, hemocytes play a central role in immunity, identifying and destroying pathogens through phagocytosis, oxidative stress, apoptosis, and autophagy – functions that can be characterized using flow cytometry and molecular analyses (37–43). Transcriptome analyses in oysters primed with Poly (I:C) have identified several pattern recognition receptors (PRRs) involved in antiviral signaling. These include, for example, retinoic acid-inducible-gene-I and Toll-like-receptors homologs of the Jak-Stat pathway, stimulator of interferon genes, interferon regulatory factors, and many IFN-stimulated genes (i.e., Viperin or ADAR), which are all implicated in the detection of virus and antiviral functions (44–48).

We aim to evaluate the potency of inactivated OsHV-1 preparations (antigens) to elicit an antiviral response in *C. gigas* hemocytes. This research is undertaken in the context of developing new strategies of immune priming to improve oyster resilience to POMS.

## 2 Material and methods

### 2.1 Preparation of OsHV-1 antigens

**Figure 1:**
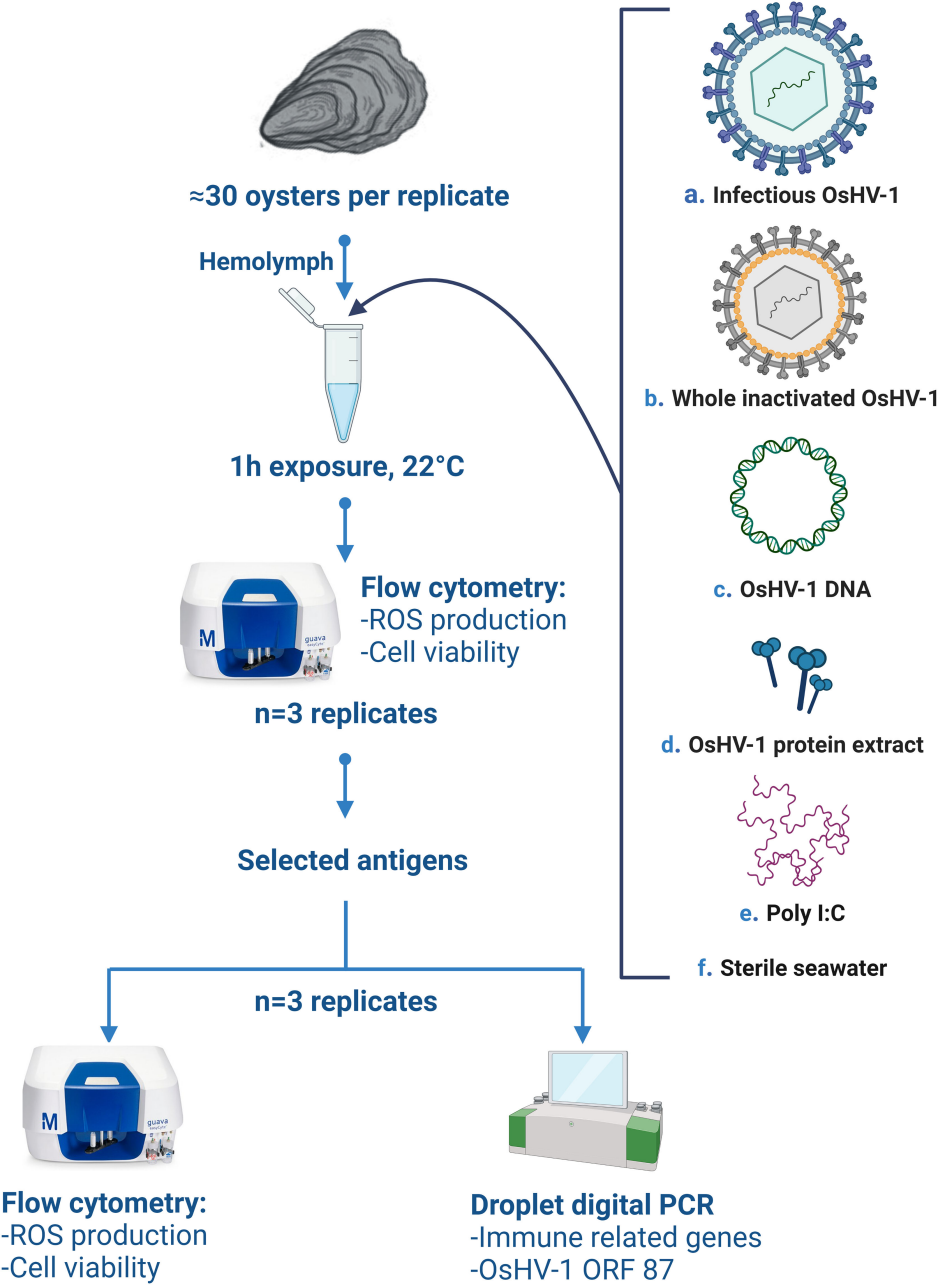
Schematic overview of the *in-vitro* experimental design, **(A)** refers to the live infectious OsHV-1, **(B)** whole virus killed by chemical (BEI, formaldehyde), heat inactivated, or exposed to freeze-thaw cycles, **(C)** OsHV-1 DNA, **(D)** OsHV-1 whole protein extract, **(E)** Poly (I:C, positive control), **(F)** sterile filtered seawater (negative control). ROS stands for ‘reactive oxygen species’, ORF to ‘open reading frame’. Figure was created using BioRender.com.

In June 2021, the experiment was conducted to (1) screen multiple preparations of inactivated OsHV-1 (hereafter referred to as antigens) by measuring immune-related functions of hemocytes using flow cytometry (FCM, repeated three times), and (2) confirm the potency of a selected subset of antigen preparations to stimulate immunity using FCM (cytotoxicity and ROS) and molecular analyses (immune- related gene expression, repeated three times, **Figure 1**).

#### 2.1.1 OsHV-1 stock

The OsHV-1 suspension stock was produced in October 2019, as described in Camara et al. (18), from diseased oysters infected with OsHV-1 during a lab challenge (49). Briefly, tissue from high virus load oysters was homogenized, purified by serial filtrations down to 0.22 μm and cryopreserved according to Kirkland et al. (50). On the 3 June 2021, cryopreserved OsHV-1 stock was defrosted by dipping in a 22°C water bath for 10 min. Viral suspension was titrated using qPCR (51) and diluted in 0.22 µm-filtered sterile seawater (SSW) to reach a final concentration of 9.0.10^5^ copies. µl^-1^. Prepared virus was then inactivated using the methods described below.

#### 2.1.2 BEI inactivation

A 0.2M Binary ethylenimine (BEI) solution was prepared by cyclization of 0.2M 2-bromoethylamin-HBr in 0.2M NaOH at 37°C for 1 h (52). The BEI solution was added to OsHV-1 suspension to either a concentration of 0.1% (v/v) and incubated at 22°C for 1 h, 4 h, or 6 h, or to a concentration of 0.04% (v/v), and incubated at 22°C for 4 h, 6 h, 9 h, 18 h, or 22 h. Inactivation reactions were stopped by addition of sodium thiosulphate 1M (neutralizing agent) to reach a final concentration of 10% (v/v). Obtained inactivated viral suspensions were stored at 4°C until use. Suspension of neutralized 0.1% (v/v) BEI diluted in filtered (0.22 µm) SSW was used as the vehicle control (VC), (**Supplementary Table 1**).

#### 2.1.3 Formaldehyde inactivation

Formaldehyde solution 37% (w/v) was added to the OsHV-1 suspension to a final concentration of 5%, 0.3%, or 0.01%, and incubated at 22°C for 2 h, 4 h, or12 h (5%); for 4 h, 8 h, 12 h, 24 h, 48 h, or 60 h (0.3%); or for 12 h, 24 h, or 60 h (0.01%), respectively. Virus inactivation was stopped by addition of 35% sodium bisulphite to reach a final concentration of 0.035%, and suspensions were then stored at 4°C. Suspension of 5% (v/v) neutralized formalin diluted in filtered (0.22 µm) SSW was used as the VC (**Supplementary Table 1**).

#### 2.1.4 Heat inactivation

For preparation of heat-inactivated OsHV-1, viral suspensions were incubated for 1 h at 45°C, 50°C, or 52°C, or for 30 min at 54°C, 56°C, and 60°C using a dry bath, and then stored at 4°C until use (**Supplementary Table 1**).

#### 2.1.5 Freeze-thaw cycles

Viral suspensions were placed at -80°C for 12 h, transferred to -20°C for 12 h, and then maintained at 4°C. After complete thawing, viral suspensions were immediately re-frozen at -80°C for 12 h. The freeze-thaw cycles were repeated twice (for Thawing 1) or three times (for Thawing 2), and the antigen suspensions were stored at 4°C until use (**Supplementary Table 1**).

#### 2.1.6 OsHV-1 DNA

Total viral DNA was extracted from 1 ml of the OsHV-1 stock suspension using blood and tissues kit (QIAGEN) according to the manufacturer’s protocol. Extracted DNA was resuspended in 10 mM Tris-HCl buffer (pH 7.4) to reach a final concentration of 10 ng DNA µl^-1^ and stored at -20°C until use. The solution 10 mM Tris-HCl buffer (pH 7.4) was used as a VC (**Supplementary Table 1**).

#### 2.1.7 Viral proteins

Total viral proteins were extracted from 5 ml of the OsHV-1 stock suspension *via* bead beating for 10 min at 1500 rpm and 4°C using a 1600 MiniG automated tissue homogenizer (SPEX Sample Prep, Metuchen, NJ). Proteins were then solubilized for 45 min by adding 2 ml of extraction buffer (100 mM potassium phosphate, 50 mM NaCl, 0.1 mM EDTA-Na2, 1% polyvinyl pyrrolidone, 2 mM phenylmethylsulfonyl fluoride and 0.1% TritonX-100; pH 7.5 at 4°C). Solubilized proteins were extracted by centrifugation at 13,500 rpm for 15 min at 4°C and ultra-filtered (10 kDa molecular weight cut-off, Amicon Ultra-0.5 10K, Merk-Millipore, Burlington, USA) following manufacturer’s specifications. The semi-purified proteins were reconstituted in 200 µl of sterile Phosphate Buffer Saline (PBS). Total protein content of the lysate was quantified by the Lowry protein assay (53), diluted in sterile PBS to reach a final concentration of 0.2 mg.ml^-1^, and stored at -80°C until use. Protein extracts were either pure (Protein 1) or diluted at 1:10 (v/v) in PBS (Protein 2). Sterile PBS was used as a vehicle control (**Supplementary Table 1**).

### 2.2 Oysters

The experiment was performed using hemolymph of hatchery-bred juvenile *C. gigas* [8 months old, mean live weight 6.7 ± 3.1 g]. These oysters were the offspring of naïve wild stocks and therefore were expected to be highly susceptible to POMS. Prior to sampling, oysters were maintained in flow-through seawater (10 µm filtered) at ambient conditions (10 – 22°C and a salinity of 35 ± 1) and fed ad libitum with hatchery-grown algal food. Experimental oysters were considered naïve to POMS/OsHV-1 due to their rearing with continuous supply of UV-sterilized seawater (80 mJ cm^-2^) and maintenance under strict biosecurity management to ensure they remained OsHV-1-free. This status was confirmed prior to the experiment by the absence of significant mortality and OsHV-1 DNA detection in tissue (n = 10) using qPCR (51). Oysters were starved for 24 h prior to hemolymph collection to minimize algal contamination of the hemolymph.

### 2.3 Hemolymph collection

Hemolymph was sampled from between 22 and 35 oysters collected and pooled daily for experimental exposure. A small notch was made in the shell using wire cutters, and oysters were bled from the adductor muscle sinus using a 25G 1.5-inch needle with 1 mL syringe, previously kept on ice. Between 150 and 1500 µl of hemolymph was withdrawn from each individual and immediately added to a 1.5 mL Eppendorf, previously kept on ice. Individual samples were checked under a light microscope (40X magnification) to confirm purity. When ≈16 mL of pure, clean hemolymph had been collected from multiple individuals, hemolymph samples were pooled, diluted (1:4 v/v) with autoclaved 0.2 µm-filtered sterile seawater (FSSW) for flow cytometry or kept undiluted for molecular analyses, and stored on ice until exposure.

### 2.4 *In-vitro* exposure

Before experimental exposure, each antigen preparation and vehicle control were diluted (1:100 v/v) with FSSW. Polyinosinic-polycytidylic acid [Poly (I:C)], a synthetic analogue of double-stranded RNA (dsRNA), was used as positive control (0.05 mg. ml^-1^ in SSW; 54) and FSSW was used as a negative control. For flow cytometry, antigens preparations or controls were added (1:80 v/v) to (1:4 v/v) diluted hemolymph (detailed above) and incubated at room temperature (22°C) for 1 h. The expression of five immune-related genes and the viral gene ORF 87 was evaluated by adding 20 µl of the diluted antigen preparations (1:100 (v/v) or controls to 400 µl of pure hemolymph in 1.5 ml Eppendorf tubes and incubated at 22°C for 1 h.

Following experimental exposure, hemocyte reactive oxygen species (ROS) production and viability were determined (detailed below). This process was repeated daily for 3 consecutive days to obtain n = 3 independent replicates.

Based on ROS production in hemocytes and an absence of cytotoxicity, a subset of promising antigens was selected for further FCM validation and molecular analyses in a second experiment. Specific antigen preparations which induced significantly less ROS production were also included in the second experiment to maintain a range of contrasted immune responses and improve validation. This second *in vitro* challenge was repeated daily for 3 consecutive days to obtain n = 3 independent replicates.

### 2.5 FCM assessments

Hemolymph samples that had been exposed to different antigen preparations for 1 h were analyzed using a Guava^®^ EasyCyte™ 5HT flow cytometer equipped with a blue laser (488 nm) and green (525/30 nm), yellow (583/26 nm), and red (695/50) detectors (EMD Millipore, USA). Samples were mixed at medium speed and acquired at a flow rate of 0.24 µL s^-1^ for 30s.

The production of intracellular ROS was measured using 2’,7’-Dichlorodihydro-fluorescein diacetate (DCFH-DA, Sigma Aldrich, D6883) according to Donaghy et al. (55). Following 30 min of experimental treatments incubation, DCFH-DA was added at a final concentration of 10 µMand incubated in the dark at room temperature (22°C) for another 30 min until analysis (= total 1 h exposure to treatments). Relative ROS production was expressed as the level of green (FL1) fluorescence.

The viability of hemocytes was measured using Fluorescein diacetate (FDA, Invitrogen, F1303) according to Rolton et al. (56). Following 50 min of experimental treatments incubation, FDA was added at a final concentration of 1.25 mg L^-1^ and incubated in the dark at room temperature for 10 min until analysis (= total 1 h exposure to treatments). Hemocytes were divided into those with high FL1 (corresponding to metabolically active/viable cells) and those with low green fluorescence (non-viable).

### 2.6 Molecular analyses

RNA was extracted from 400 µl of hemolymph previously exposed to 20 µl of antigen preparation, SSW (negative control), Poly I:C (positive control), or live infectious OsHV-1 using the Quick RNA/DNA Miniprep plus kit (Zymo Research) according to the manufacturer’s protocol. RNA was eluted in 50 µl DNAse/RNase-free water. As described in Delisle et al. (49), samples were treated with DNAse I (TURBOTM DNase, Invitrogen), the absence of DNA in the samples was confirmed by a 16S PCR assay, purity of the isolated RNA was assessed, and DNAse-treated RNA was transcribed into cDNA. Finally, droplet digital PCR (ddPCR) was conducted in an automated droplet generator (QX200 Droplet Digital PCR SystemTM, Bio-Rad) to determine the expression of five genes (Jak, Stat6, Viperin, IRF2, Myd88) related to oyster innate immunity (44, 57, 58), as well as the ORF 87, an OsHV-1 gene selected from the 39 ORFs described by Segarra et al. (59). Each ddPCR reaction included 1 µl of 3 µM of the primers (Jak, Stat, Viperin, Myd88) or 10 µM (IRF2, ORF87), 10 µl ddPCR Supermix for Evagreen (Bio-Rad), 2 µl cDNA, and 8 µl sterile water for a total reaction volume of 21 µl. As described in Delisle et al. (49), ddPCR was performed using the following cycling protocol: hold at 95°C for 5 s, 45 cycles of 95°C for 30 s, 60°C for 1 min, 4°C for 5 min, and a final enzyme deactivation step at 90°C for 5 min. The plate was then analyzed on the QX200 instrument (Bio-Rad). For each ddPCR plate run, at least one negative control (RNA/DNA-free water; Life Technologies), and one positive control (*C. gigas* DNA or Gblock for ORF87 diluted 1/10,000) were included.

### 2.7 Statistical analyses

Statistical analyses were computed using R 4.2.1 (https://www.r-project.org/) and the packages ‘ggpubr’ (60) and ‘rstatix’ (61). One way ANOVA and t tests were performed to evaluate the effects of each antigen preparation on ROS production, hemocytes viability, and gene expression, in comparison to the effects of SSW exposure (negative control); p-values were adjusted with Holm correction. For gene expression, a heatmap was constructed using Multiple Experiment Viewer software (62; http://mev.tm4.org/#/datasets/upload). For all analyses, the threshold significance level was set at 0.05.

### 2.8 Ethics approval

The study was conducted according to the guidelines of the Declaration of Helsinki and approval of the Animal Ethics Committee was not applicable for the use of oysters.

## 3 Results

### 3.1 79% of the antigenic preparations did not affect the viability of oyster hemocytes

**Figure 2:**
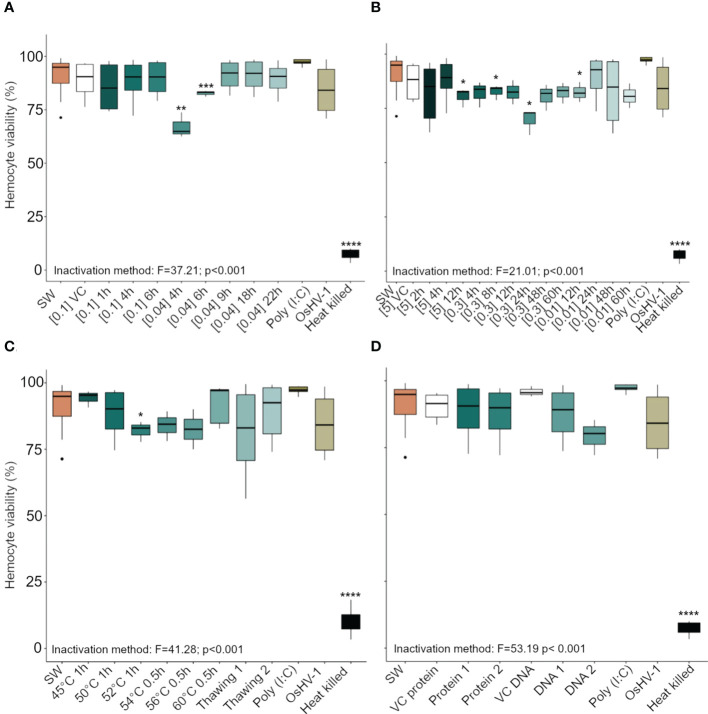
Viability (in percent) of *Crassostrea gigas* hemocytes exposed for 1 h to OsHV-1, which had been inactivated with different preparations of **(A)** Binary ethylenimine BEI, **(B)** formaldehyde, and **(C)** temperature treatments. **(D)** shows viability of hemocytes exposed to two viral protein concentrations and purified viral DNA (see Supplementary Table 1 for details of the different treatments). Box plots indicate the median, upper, and lower quartiles. Whiskers indicate the highest and the lowest percent viable hemocytes and dots indicate outliers. Significance levels are expressed by asterisks: * (p < 0.05), ** (p < 0.01), ***(p < 0.001), **** (p < 0.0001). N = 3/6 replicates. ‘SW’ stands for seawater, ‘VC’ for vehicle control.

Of the 33 antigenic preparations tested, 26 had no cytotoxic effect on oyster hemocytes (as measured by viability). However, hemocytes exposed for 1 h to the following antigen preparations – OsHV-1 previously inactivated using BEI 0.04% for 4 h and 6 h (**Figure 2A**), formaldehyde 5% for 12 h, formaldehyde 0.3% for 8 h or 24 h, or formaldehyde 0.01% for 12 h (**Figure 2C**), and heated at 52°C for 1 h (**Figure 2D**) – showed a significant reduction in viability compared to hemocytes exposed to SSW. The viability of hemocytes that had been heat killed (negative control) was very low (10.2 ± 5.2% mean ± SD, n = 3) compared to hemocytes exposed to SSW (91.3 ± 8.0%, p = 1.7e-13) (**Figure 2**).

### 3.2 Antigenic preparations induced ROS production in hemocytes

**Figure 3:**
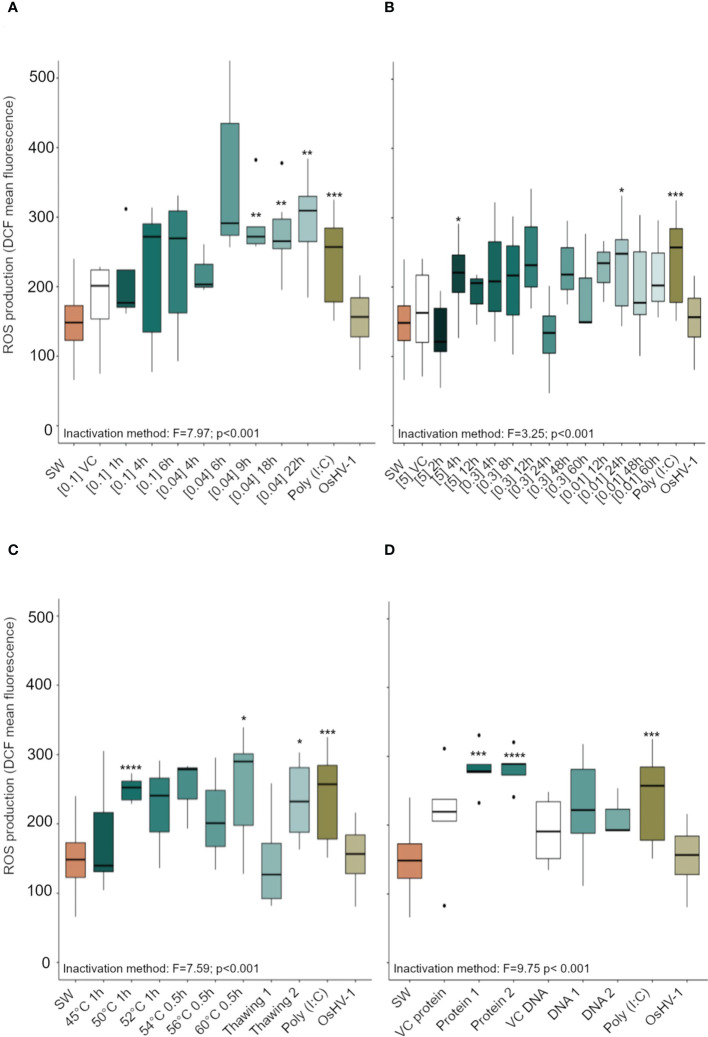
Reactive oxygen species (ROS) production (expressed in arbitrary unit, a.u) in *Crassostrea gigas* hemocytes exposed for 1 h to OsHV-1, which had been inactivated with different preparations of **(A)** Binary ethylenimine BEI, **(B)** formaldehyde, and **(C)** temperature treatments. **(D)** shows ROS production in hemocytes exposed to two viral protein concentrations and purified viral DNA (see Supplementary Table 1 for details of the different treatments). N = 3/6 replicates, box plots indicate the median, upper and lower quartiles; whiskers indicate the highest and the lowest values of the dataset and dots indicates outliers. Significance levels are expressed by asterisks: * (p < 0.05), ** (p<0.01), ***(p<0.001), **** (p<0.0001). ‘SW’ stands for seawater, ‘VC’ for vehicle control.

Hemocytes of C. gigas that had been exposed to 10 antigenic preparations of inactivated OsHV-1 and to the positive control (Poly I:C) showed increased ROS production (**Figure 3**). Specifically, preparations of virus inactivated using BEI 0.04% at 9, 18, and 22 h (**Figure 3A**), formaldehyde 5% for 4 h, formaldehyde 0.01% for 24 h (**Figure 3B**), heat shock at 50°C for 1 h, at 60°C for 0.5 h, or three freeze-thaw cycles (**Figure 3C**), as well as viral protein extracts pure or diluted 1/10 (v/v, **Figure 3D**), all significantly increased hemocyte ROS production compared to those exposed to SSW (p < 0.001).

Based on an absence of cytotoxicity and high levels of ROS production (using FCM), 10 antigen preparations, as well as eight additional antigen preparations that induced a limited subcellular immune response (**Figures 2**, **3**), were selected for validation (using FCM and molecular analysis).

### 3.3 All the selected antigen preparations induced upregulation of immune-related genes

**Figure 4:**
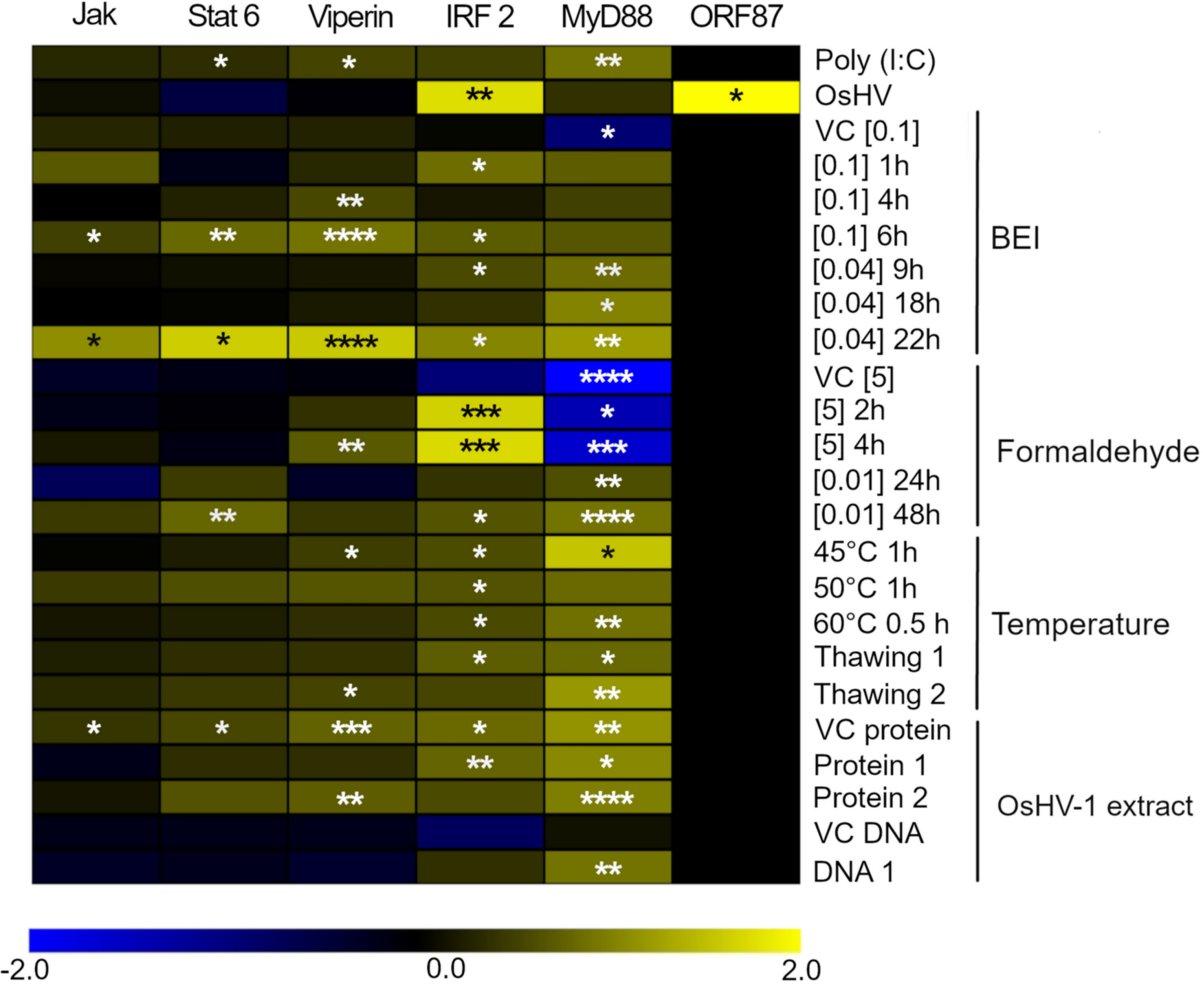
Heatmap focusing on the expression of 5 genes related to the innate immune response in *Crassostrea gigas* and ORF87, an OsHV-1 ORF expressed early after infection. The intensities of the colors indicate the magnitude of the differential expression (log2 fold-change), N = 3 replicates. The fold-changes were calculated by comparing the expression of each gene in hemocytes exposed to Poly (I:C), live infectious OsHV-1, or OsHV-1 inactivated, with its expression in negative control (hemocytes exposed to SSW). Significance levels are expressed by asterisks: * (p < 0.05), ** (p < 0.01), ***(p < 0.001), **** (p < 0.0001).

All the selected antigen preparations induced the upregulation of at least one of the immune- related genes of *C. gigas*. Exposure of hemocytes to the Poly (I:C) at 0.05 mg. ml^-1^ for 1 h induced a significant upregulation of MyD88, Viperin, and Stat6 (**Figure 4**). Exposure of hemocytes to OsHV-1, which had been inactivated using BEI 0.04% for 22 h, resulted in a significant upregulation of all five of the immune- related genes analyzed. Vehicle controls did not induce the expression of the immune- related genes in hemocytes, except the phosphate buffered saline (VC protein, **Supplementary Table 1**), which induced a significant upregulation of Stat6, Viperin, IRF2, and MyD88. As expected, the expression of ORF87 was only detected in hemocytes exposed to live infectious OsHV-1 (**Figure 4**).

## 4 Discussion

In the present study, we were able to stimulate an antiviral response in juvenile *Crassostrea gigas* hemocytes using inactivated OsHV-1 and viral extracts, as shown by ROS induction and upregulation of antiviral response-related genes. A previous study, in which *C. gigas* hemocytes were exposed to live OsHV-1, also resulted in the expression of genes involved in immune-related functions (42); however, this study is the first time inactivated OsHV-1 preparations have been shown to elicit an immune response *in-vitro*.

Virus inactivation transforms antigens from being infectious to non-infectious, and it is important to determine any cytotoxicity of the antigen preparations prior to determining their potency (52). In the absence of bivalve cell line cultures in which to determine cytotoxicity, here, we used a FCM-based assessment of hemocyte viability for rapid ***in-vitro*** screening of the OsHV-1 inactivated preparations. Morga et al. (63), similarly used FCM to determine hemocyte viability following an ***in-vitro*** exposure of *C. gigas* hemocytes to the protozoan parasite *Bonamia ostreae*. Among the 33 antigen preparations tested in our study, only seven induced a significant decline in hemocyte viability. Incomplete neutralization of the denaturing agent could explain this observed cytotoxicity. The residual infectivity post-inactivation of the preparations tested was verified by the absence of the open reading frame 87 (ORF87) expression in the hemocytes following 1h of antigen exposure. This ORF codes for an apoptosis inhibitor protein and is expressed during the first few hours post OsHV-1 infection in oysters (42, 59).

A 1-hour exposure of hemocytes to 0.5 mg.ml^-1^ of Poly (I:C) was sufficient to induce cellular ROS production – a proxy of immune response in bivalves (64) – and an upregulation of the genes coding key antiviral effectors: MyD88, Stat 6, and Viperin, validating our screening approach. Increased ROS indicates a stimulation of an immune response, as ROS production is associated with internal chemical destruction of engulfed pathogens or foreign particles within hemocytes (65, 66). The mechanisms of hemocyte activation in oyster immune defense, however, are still largely unknown. In response to foreign particles, oyster hemocytes can: secrete effectors extracellularly; phagocytose particles, where they are destroyed by ROS and defense molecules stored in granules; and following stress or recognition of foreign particles by soluble and cellular pattern recognition receptors, induce the expression of specific immune genes (67). An induction of MyD88 gene, an essential signal transducer in the interleukin-1 and Toll-like receptor signaling pathways, was also observed in mussel (*Mytilus galloprovincialis*) and scallop (*Pecten maximus*) hemocytes that had been stimulated for 8 h and 3 h, respectively, with 50 µg.ml^-1^ of Poly (I:C), a much higher concentration than was used in the present study (68, 69). Upregulation of Viperin – an interferon-inducible antiviral protein – and Stat 6 – a signal transducer and activator of transcription – have also been observed in oyster hemocytes primed with Poly (I:C), in comparison to those exposed to seawater (46, 70).

The interaction between OsHV-1 and the oyster host cells has not been fully elucidated, and the functions of proteins coded by OsHV-1 genome are largely unknown. However, antibody blocking and pull-down assays suggest the potential implication of three putative OsHV-1 membrane proteins (ORF 25, ORF 41, and ORF 72) in the virus/host interaction by binding of host cytoskeleton (71, 72). Interestingly, antigen preparations resulting from BEI inactivation induced the strongest immune and antiviral response. Binary ethylenimine is an aziridine preparation commonly used in veterinary vaccine production as an inactivating agent (73). At 1 mM, BEI induces an alkylation of the nucleic acids without damaging proteins (74). In the present work, high ROS production coupled with upregulation of the five tested genes were recorded when using OsHV-1 inactivated with BEI at [0.04%] for 22h, supporting the role of viral proteins in the stimulation of antiviral response in oysters. Conversely, we observed a reduced potency of antigen preparations that were inactivated by formaldehyde, one of the most widely used inactivating agents, for an extended exposure time (12 and 48h), suggesting an irreversible denaturation of proteins induced by formaldehyde (75).

Three antigen preparations inactivated with BEI induced a strong upregulation of the transcript coding for Viperin. Viperin is a highly conserved evolutionary host protein (76), which restricts the replication of a range of RNA and DNA viruses [e.g., human cytomegalovirus (77), immunodeficiency virus (78), and Hepatitis C virus (79)], by interacting with viral protein and altering the site of virus budding. In *C. gigas*, Viperin has been reported to be one of the earliest and most regulated genes in response to OsHV-1 exposure (54, 80), and it also exhibits the same level of antiviral activity as human Viperin against Dengue virus ***in-vitro*** (46, 80). Consequently, its expression in hemocytes could constitute a good indicator of the potency of the antigen preparations.

Besides chemical agents, physical methods were used to inactivate OsHV-1 in this study. Thermal inactivation of OsHV-1 at 50°C and 60°C and long thawing cycles caused an increase in ROS production, while the associated molecular responses appeared moderate. These variations in the results could probably be explained by the broad range of stressors able to induce ROS production in bivalves (81), in comparison to the specificity of the antiviral response. Nonetheless, we obtained a good correlation overall between immune-related markers measured *via* flow cytometry (ROS production) and qPCR analyses (immune-gene expression), with the ranking of the 10 best antigen preparations maintained when upregulation of immunity genes was considered.

Of note, protein extracts obtained from OsHV-1 and resuspended in PBS also induced a marked positive expression of MyD88, IRF2, and Viperin; however, the confounding effect of the vehicle control PBS on the hemocytes cannot be excluded. Similar induction of immune response following PBS exposure has been reported (82, 83).

To conclude, these findings are significant as they evidence for the first time the potential for stimulating oyster’s innate immunity using OsHV-1 antigens, which may enable cost-effective therapeutic treatment to mitigate the economic impacts of OsHV-1/POMS. For instance, chemical inactivation using the Binary ethylenimine at 0.04% for 22 h was identified as the best candidate preparation requiring additional research. However, it is essential to test these antigen preparations further using *in-vivo* infection models to validate promising candidate pseudo-vaccines. Nonetheless, we demonstrated that the use of flow cytometry-based cellular assays was an effective and rapid screening tool to select treatments for ‘pseudo-vaccine’ development.

## Data availability statement

The original contributions presented in the study are included in the article/**Supplementary Materials**. Further inquiries can be directed to the corresponding authors.

## Author contributions

LD, AR, JV, conception and design. LD and AR lab trial and data analyses. All authors, writing, revision/editing. LD, JV funding. All authors contributed to the article and approved the submitted version.

## References

[1] Lafferty KD Porter JW Ford SE. Are diseases increasing in the ocean? Annu Rev Ecol Evol Syst (2004) 35:31–54. doi: 10.1146/annurev.ecolsys.35.021103.105704

[2] Burge CA Mark Eakin C Friedman CS Froelich B Hershberger PK Hofmann EE. Climate change influences on marine infectious diseases: implications for management and society. Ann Rev Mar Sci (2014) 6:249–77. doi: 10.1146/annurev-marine-010213-135029 23808894

[3] Harvell CD Montecino-Latorre D Caldwell JM Burt JM Bosley K Keller A. Disease epidemic and a marine heat wave are associated with the continental-scale collapse of a pivotal predator (Pycnopodia helianthoides). Sci Adv (2019) 5:1–9. doi: 10.1126/sciadv.aau7042 PMC6353623 30729157

[4] Daszak P Cunningham AA Hyatt AD. Emerging infectious diseases of wildlife - threats to biodiversity and human health. Science (2000) 80:443–4. doi: 10.1126/science.287.5452.443 10642539

[5] Wiethoelter AK Beltrán-Alcrudo D Kock R Mor SM. Global trends in infectious diseases at the wildlife-livestock interface. Proc Natl Acad Sci USA (2015) 112:9662–7. doi: 10.1073/pnas.1422741112 PMC4534210 26195733

[6] The International Bank for Reconstruction and Development. Reducing disease risk in aquaculture world bank report number 88257-GLB. (Washington, DC, USA: World Bank) (2014). doi: 10.13140/RG.2.1.4525.5529.

[7] Barbosa-Solomieu V Dégremont L Vázquez-Juárez R Ascencio-Valle F Boudry P Renault T. Ostreid herpesvirus 1 (OsHV-1) detection among three successive generations of pacific oysters (*Crassostrea gigas*). Virus Res (2005) 107:47–56. doi: 10.1016/j.virusres.2004.06.012 15567033

[8] EFSA. Scientific opinion on the increased mortality events in pacific oysters. Eur Food Saf (2010) 8:1–60. doi: 10.2903/j.efsa.2010.1894 PMC13129988 42079548

[9] EFSA. Oyster mortality. EFSA J (2015) 13:4122. doi: 10.2903/j.efsa.2015.4122

[10] Pernet F Lupo C Bacher C Whittington RJ. Infectious diseases in oyster aquaculture require a new integrated approach. Philos Trans R Soc B Biol Sci (2016) 371. doi: 10.1098/rstb.2015.0213 PMC4760143 26880845

[11] De Lorgeril J Lucasson A Petton B Toulza E Montagnani C Clerissi C. Immune-suppression by OsHV-1 viral infection causes fatal bacteraemia in pacific oysters. Nat Commun (2018) 9:4215. doi: 10.1038/s41467-018-06659-3 30310074 PMC6182001

[12] Segarra A Baillon L Faury N Tourbiez D Renault T. Detection and distribution of ostreid herpesvirus 1 in experimentally infected pacific oyster spat. J Invertebr Pathol (2016) 133:59–65. doi: 10.1016/j.jip.2015.11.013 26674009

[13] Jenkins C Hick P Gabor M Spiers Z Fell S Gu X. Identification and characterization of an ostreid herpesvirus-1 microvariant (OsHV-1 μ-var) in *Crassostrea gigas* (Pacific oysters) in Australia. Dis Aquat Organ (2013) 105:109–26. doi: 10.3354/dao02623 23872855

[14] Keeling SE Brosnahan CL Williams R Gias E Hannah M Bueno R. New Zealand juvenile oyster mortality associated with ostreid herpesvirus 1-an opportunistic longitudinal study. Dis Aquat Organ (2014) 109:231–9. doi: 10.3354/dao02735 24991849

[15] Hwang JY Park JJ Yu HJ Hur YB Arzul I Couraleau Y. Ostreid herpesvirus 1 infection in farmed pacific oyster larvae *Crassostrea gigas* (Thunberg) in Korea. J Fish Dis (2013) 36:969–72. doi: 10.1111/jfd.12093 23957681

[16] Burge CA Friedman CS Kachmar ML Humphrey KL Moore JD Elston RA. The first detection of a novel OsHV-1 microvariant in San Diego, California, USA. J Invertebr Pathol (2021) 184:107636. doi: 10.1016/j.jip.2021.107636 34116033

[17] Dégremont L Nourry M Maurouard E. Mass selection for survival and resistance to OsHV-1 infection in *Crassostrea gigas* spat in field conditions: response to selection after four generations. Aquaculture (2015) 446:111–21. doi: 10.1016/j.aquaculture.2015.04.029

[18] Camara MD Yen S Kaspar HF Kesarcodi-Watson A King N Jeffs AG. Assessment of heat shock and laboratory virus challenges to selectively breed for ostreid herpesvirus 1 (OsHV-1) resistance in the pacific oyster, *Crassostrea gigas* . Aquaculture (2017) 469:50–8. doi: 10.1016/j.aquaculture.2016.11.031

[19] Divilov K Schoolfield B Morga B Dégremont L Burge CA Mancilla Cortez D. First evaluation of resistance to both a California OsHV-1 variant and a French OsHV-1 microvariant in pacific oysters. BMC Genet (2019) 20:96. doi: 10.1186/s12863-019-0791-3 31830898 PMC6909534

[20] Gutierrez AP Symonds J King N Steiner K Bean TP Houston RD. Potential of genomic selection for improvement of resistance to ostreid herpesvirus in pacific oyster (*Crassostrea gigas*). Anim Genet (2020) 51:249–57. doi: 10.1111/age.12909 31999002

[21] Witteveldt J Cifuentes CC Vlak JM Van Hulten MCW. Protection of *Penaeus monodon* against white spot syndrome virus by oral vaccination. J Virol (2004) 78:2057–61. doi: 10.1128/JVI.78.4.2057 PMC369486 14747570

[22] Dadar M Dhama K Vakharia VN Hossein S Karthik K Tiwari R. Advances in aquaculture vaccines against fish Pathogens : global status. Rev Fish Sci Aquac (2016) 0:1–34. doi: 10.1080/23308249.2016.1261277

[23] Virol A Feng S. Recent progress in the development of white spot syndrome virus vaccines for protecting shrimp against viral infection. Arch Virol (2017) 10:2923–36. doi: 10.1007/s00705-017-3450-x 28647845

[24] Bøgwald J Dalmo RA. Review on immersion vaccines for Fish : an update 2019. Microorganisms (2019) 7:627. doi: 10.3390/microorganisms7120627 31795391 PMC6955699

[25] Yang W Tran NT Zhu C Zhang M Yao D Aweya JJ. Enhanced immune responses and protection against the secondary infection in mud crab (*Scylla paramamosain*) primed with formalin-killed *Vibrio parahemolyticus* . Aquaculture (2020) 529:735671. doi: 10.1016/j.aquaculture.2020.735671

[26] Buchmann K. Evolution of innate immunity: clues from invertebrates *via* fish to mammals. Front Immunol (2014) 5:459. doi: 10.3389/fimmu.2014.00459 25295041 PMC4172062

[27] Netea MG Domínguez- Andrés J Barreiro LB Chavaki T Divangahi M Fuchs E. Defining trained immunity and its role in health and disease. Nat Rev Immunol (2020) 20:375–88. doi: 10.1038/s41577-020-0285-6 PMC7186935 32132681

[28] Netea MG Quintin J van der Meer JWM. Trained immunity: a memory for innate host defense. Cell Host Microbe (2011) 9:355–61. doi: 10.1016/j.chom.2011.04.006 21575907

[29] Milutinović B Kurtz J. Immune memory in invertebrates. Semin Immunol (2016) 28:328–42. doi: 10.1016/j.smim.2016.05.004 27402055

[30] Sheehan G Farrell G Kavanagh K. Immune priming: the secret weapon of the insect world. Virulence (2020) 1:238–46. doi: 10.1080/21505594.2020.1731137 PMC7051127 32079502

[31] Yao T Lu J Bai C Xie Z Ye L. The enhanced immune protection in small abalone *Haliotis diversicolor* against a secondary infection with *Vibrio harveyi* . Front Immunol (2021) 12:685896. doi: 10.3389/fimmu.2021.685896 34295333 PMC8290317

[32] Cong M Song L Wang L Zhao J Qiu L Li L. The enhanced immune protection of zhikong scallop chlamys farreri on the secondary encounter with listonella anguillarum. Comp Biochem Physiol Part B Biochem Mol Biol (2008) 151:191–6. doi: 10.1016/j.cbpb.2008.06.014 18652907

[33] Zhang T Qiu L Sun Z Wang L Zhou Z Liu R. The specifically enhanced cellular immune responses in pacific oyster (*Crassostrea gigas*) against secondary challenge with *Vibrio splendidus* . Dev Comp Immunol (2014) 45:141–50. doi: 10.1016/j.dci.2014.02.015 24607288

[34] Lafont M Petton B Vergnes A Pauletto M Segarra A Gourbal B. Long-lasting antiviral innate immune priming in the lophotrochozoan pacific oyster, *Crassostrea gigas* . Sci Rep (2017) 7:1–14. doi: 10.1038/s41598-017-13564-0 29030632 PMC5640609

[35] Lafont M Vergnes A Vidal-dupiol J De Lorgeril J Gueguen Y Haffner P. A sustained immune response supports long-term antiviral immune priming in the pacific oyster, *Crassostrea gigas* . Host Microbe Biol (2020) 11:1–17. doi: 10.1128/mBio.02777-19 PMC7064767 32156821

[36] Fallet M Montagnani C Petton B Dantan L Lorgeril J Comarmond S. Early life microbial exposures shape the *Crassostrea gigas immune* system for lifelong and intergenerational disease protection. Microbiome (2022) 1:1–21. doi: 10.1186/s40168-022-01280-5 PMC9167547 35659369

[37] Cheng T. Hemocytes: forms and functions. In: The Eastern oyster crassostrea virginica. USA: College Park, MD (1996). p. 299–333.

[38] Allam B Ford SE. Effects of the pathogenic *Vibrio tapetis* on defence factors of susceptible and non-susceptible bivalve species: haemocyte changes following *in vitro* challenge. Fish Shellfish Immunol (2006) 20:374–83. doi: 10.1016/j.fsi.2005.05.012 16023865

[39] Labreuche Y Lambert C Soudant P Boulo V Huvet A Nicolas J. Cellular and molecular hemocyte responses of the pacific oyster, *Crassostrea gigas*, following bacterial infection with *Vibrio aestuarianus* . Microbes Infect (2006) 8:2715–24. doi: 10.1016/j.micinf.2006.07.020 16978900

[40] Allam B Raftos D. Immune responses to infectious diseases in bivalves. J Invertebr Pathol (2015) 131:121–36. doi: 10.1016/j.jip.2015.05.005 26003824

[41] Buchmann JP Holmes EC. Cell walls and the convergent evolution of the viral envelope. Microbiol Mol Biol Rev (2015) .79:403–18. doi: 10.1128/MMBR.00017-15.Address PMC4651029 26378223

[42] Morga B Faury N Guesdon S Chollet B Renault T. Haemocytes from *Crassostrea gigas* and OsHV-1: a promising *in vitro* system to study host/virus interactions. J Invertebr Pathol (2017) 150:45–53. doi: 10.1016/j.jip.2017.09.007 28911815

[43] Picot S Faury N Pelletier C Arzul I Chollet B Dégremont L. Monitoring autophagy at cellular and molecular level in *Crassostrea gigas* during an experimental ostreid herpesvirus 1 (OsHV-1) infection. Front Cell Infect Microbiol (2022) 12:858311. doi: 10.3389/fcimb.2022.858311 35444958 PMC9014014

[44] Green TJ Montagnani C. Poly I: c induces a protective antiviral immune response in the pacific oyster (*Crassostrea gigas*) against subsequent challenge with ostreid herpesvirus (OsHV-1 μvar). Fish Shellfish Immunol (2013) 35:382–8. doi: 10.1016/j.fsi.2013.04.051 23685009

[45] Green TJ Raftos D Speck P Montagnani C. Antiviral immunity in marine molluscs. J Gen Virol (2015) 96:2471–82. doi: 10.1099/jgv.0.000244 26297577

[46] Green TJ Speck P Geng L Raftos D Beard MR Helbig KJ. Oyster viperin retains direct antiviral activity and its transcription occurs via a signaling pathway involving a heat-stable haemolymph protein. J Gen Virol (2015) 96:3587–97. doi: 10.1099/jgv.0.000300 26407968

[47] Wang L Song X Song L. The oyster immunity. Dev Comp Immunol (2018) 80:99–118. doi: 10.1016/j.dci.2017.05.025 28587860

[48] Agius JR Corbeil S Helbig KJ. Immune control of herpesvirus infection in molluscs. Pathogens (2020) 9:1–11. doi: 10.3390/pathogens9080618 PMC7460283 32751093

[49] Delisle L Laroche O Hilton Z Burguin J Rolton A Berry J. Understanding the dynamic of POMS infection and the role of microbiota composition in the survival of pacific oysters, *Crassostrea gigas* . Microbiol Spectr (2022) 6. doi: 10.21203/rs.3.rs-1636731/v1 PMC9769987 36314927

[50] Kirkland PD Hick P Gu X. Development of a laboratory model for infectious challenge of pacific oysters (Crassostrea gigas) with ostreid herpesvirus type-1 (2015). Available at: http://frdc.com.au/Archived-Reports/FRDCProjects/2012-052-DLD.pdf.

[51] Martenot C Oden E Travaillé E Malas JP Houssin M. Comparison of two real-time PCR methods for detection of ostreid herpesvirus 1 in the pacific oyster *Crassostrea gigas* . J Virol Methods (2010) 170:86–9. doi: 10.1016/j.jviromet.2010.09.003 20837066

[52] Bahnemann HG. Inactivation of viral antigens for vaccine preparation with particular reference to the application of binary ethylenimine. Vaccine (1990) 8:299–303. doi: 10.1016/0264-410X(90)90083-X 2204242 PMC7173316

[53] Fryer HJL Davis GE Manthorpe M Varon S. Lowry protein assay using an automatic microtiter plate spectrophotometer. Anal Biochem (1986) 153:262–6. 3706709 10.1016/0003-2697(86)90090-4

[54] Green TJ Rolland JL Vergnes A Raftos D Montagnani C. OsHV-1 countermeasures to the pacific oyster’s anti-viral response. Fish Shellfish Immunol (2015) 47:435–43. doi: 10.1016/j.fsi.2015.09.025 26384844

[55] Donaghy L Kraffe E Le Goïc N Lambert C Volety AK Soudant P. Reactive oxygen species in unstimulated hemocytes of the pacific oyster *Crassostrea gigas*: a mitochondrial involvement. PloS One (2012) 7:1–10. doi: 10.1371/journal.pone.0046594 PMC3463542 23056359

[56] Rolton A Delisle L Berry J Venter L Charles S Adams S. Flow cytometric characterization of hemocytes of the flat oyster, *Ostrea chilensis* . Fish Shellfish Immunol (2020) 97:411–20. doi: 10.1016/j.fsi.2019.12.071 31877358

[57] He Y Jouaux A Ford SE Lelong C Sourdaine P Mathieu M. Transcriptome analysis reveals strong and complex antiviral response in a mollusc. Fish Shellfish Immunol (2015) 46:131–44. doi: 10.1016/j.fsi.2015.05.023 26004318

[58] Rosani U Varotto L Domeneghetti S Arcangeli G Pallavicini A Venier P. Dual analysis of host and pathogen transcriptomes in ostreid herpesvirus 1-positive *Crassostrea gigas* . Environ Microbiol (2015) 17:4200–12. doi: 10.1111/1462-2920.12706 25384719

[59] Segarra A Faury N Pépin JF Renault T. Transcriptomic study of 39 ostreid herpesvirus 1 genes during an experimental infection. J Invertebr Pathol (2014) 119:5–11. doi: 10.1016/j.jip.2014.03.002 24681357

[60] Kassambara A. Ggpubr: “ggplot2” based publication ready plots (2020). Available at: https://cran.r-project.org/web/packages/ggpubr/index.html.

[61] Kassambara A. Rstatix: pipe-friendly framework for basic statistical tests (2021). Available at: https://cran.r-project.org/web/packages/rstatix/index.html.

[62] Saeed AI Sharov V White J Li J Liang W Bhagabati N. TM4: a free, open-source system for microarray data management and analysis. Biotechniques (2003) 34:374–8. doi: 10.2144/03342mt01 12613259

[63] Morga B Arzul I Chollet B Renault T. Infection with the protozoan parasite *Bonamia ostreae* modifies *in vitro* haemocyte activities of flat oyster *Ostrea edulis* . Fish Shellfish Immunol (2009) 26:836–42. doi: 10.1016/j.fsi.2009.03.018 19358892

[64] De la Ballina NR Maresca F Cao A Villalba A. Bivalve haemocyte subpopulations: a review. Front Immunol (2022) 13:826255. doi: 10.3389/fimmu.2022.826255 35464425 PMC9024128

[65] Cheng T. Cellular defense mechanisms in oysters. science Pu. Fingerman R Nagabhushanam M, editors. New York: Science Publisher Inc (2000).

[66] Chu FL. Defense mechanisms of marine bivalves. In: Fingerman R Nagabhushanam M, editors. Recent advances in marine biotechnology. Enfield Science Publishers Inc (2000). p. 1–42.

[67] Bachere E Rosa RD Schmitt P Poirier AC Merou N Charriere GM. The new insights into the oyster antimicrobial defense: cellular, molecular and genetic view. Fish Shellfish Immunol (2015) 46:50–64. doi: 10.1016/j.fsi.2015.02.040 25753917

[68] Pauletto M Milan M Moreira R Novoa B Figueras A Babbucci M. Deep transcriptome sequencing of *Pecten maximus* hemocytes: a genomic resource for bivalve immunology. Fish Shellfish Immunol (2014) 37:154–65. doi: 10.1016/j.fsi.2014.01.017 24486903

[69] Moreira R Romero A Rey-Campos M Pereiro P Rosani U Novoa B. Stimulation of mytilus galloprovincialis hemocytes with different immune challenges induces differential transcriptomic, miRNomic, and functional responses. Front Immunol (2020) 11:606102. doi: 10.3389/fimmu.2020.606102 33391272 PMC7773633

[70] Green TJ Helbig K Speck P Raftos DA. Primed for success: oyster parents treated with poly(I:C) produce offspring with enhanced protection against ostreid herpesvirus type I infection. Mol Immunol (2016) 78:113–20. doi: 10.1016/j.molimm.2016.09.002 27616590

[71] Martenot C Faury N Morga B Degremont L Lamy J-B Houssin M. Exploring first interactions between ostreid herpesvirus 1 (OsHV-1) and its host, *Crassostrea gigas*: effects of specific antiviral antibodies and dextran sulfate. Front Microbiol (2019) 10:1128. doi: 10.3389/fmicb.2019.01128 31178841 PMC6543491

[72] Yu J Liu Y Huang B Li C Wang D Yao M. Characterization of host cell potential proteins interacting with OsHV-1 membrane proteins. Viruses (2021) 13:1–13. doi: 10.3390/v13122518 PMC8705437 34960787

[73] Zhu F Qian X Ma X. Comparative transcriptomic analysis of crab hemocytes in response to white spot syndrome virus or *Vibrio alginolyticus* infection. Fish Shellfish Immunol (2018) 80:165–79. doi: 10.1016/j.fsi.2018.06.003 29870828

[74] Groseil C Guerin P Adamowicz P. Evaluation by polymerase chain reaction on the effect of betapropiolactone and binary ethyleneimine on DNA. Biologicals (1995) 23:213–20. doi: 10.1006/biol.1995.0035 8527120

[75] Sanders B Koldijk M Schuitemaker H. Inactivated viral vaccines,” in vaccine analysis: strategies, principles, and control. Springer (2015) p. 45–80. doi: 10.1007/978-3-662-45024-6

[76] Fitzgerald KA. The interferon inducible gene: viperin. J Interf Cytokine Res (2011) 31:131–5. doi: 10.1089/jir.2010.0127 PMC3021329 21142818

[77] Chin KC Cresswell P. Viperin (cig5), an IFN-inducible antiviral protein directly induced by human cytomegalovirus. Proc Natl Acad Sci USA (2001) 98:15125–30. doi: 10.1073/pnas.011593298 PMC64994 11752458

[78] Nasr N Maddocks S Turville SG Harman AN Woolger N Helbig KJ. HIV-1 infection of human macrophages directly induces viperin which inhibits viral production. Blood (2012) 120:778–88. doi: 10.1182/blood-2012-01-407395 22677126

[79] Helbig KJ Lau DTY Semendric L Harley HAJ Beard MR. Analysis of ISG expression in chronic hepatitis c identifies viperin as a potential antiviral effector. Hepatology (2005) 42:702–10. doi: 10.1002/hep.20844 16108059

[80] Green TJ Montagnani C Benkendorff K Robinson N Speck P. Ontogeny and water temperature influences the antiviral response of the pacific oyster, *Crassostrea gigas* . Fish Shellfish Immunol (2013) 36:151–7. doi: 10.1016/j.fsi.2013.10.026 24200990

[81] Donaghy L Hong HK Jauzein C Choi KS. The known and unknown sources of reactive oxygen and nitrogen species in haemocytes of marine bivalve molluscs. Fish Shellfish Immunol (2015) 42:91–7. doi: 10.1016/j.fsi.2014.10.030 25449373

[82] Catalán TP Wozniak A Niemeyer HM Kalergis AM Bozinovic F. Interplay between thermal and immune ecology: effect of environmental temperature on insect immune response and energetic costs after an immune challenge. J Insect Physiol (2012) 58:310–7. doi: 10.1016/j.jinsphys.2011.10.001 22019347

[83] Gálvez D Añino Y Vega C Bonilla E. Immune priming against bacteria in spiders and scorpions? PeerJ (2020) 2020:0–14. doi: 10.7717/peerj.9285 PMC7278890 32547885

