# Supplementary material for: Inactivated ostreid herpesvirus-1 induces an innate immune response in the Pacific oyster, Crassostrea gigas, hemocytes
Source: Front Immunol. 2023 Apr 28;14:1161145. doi: 10.3389/fimmu.2023.1161145 (PMC10175643; doi:10.3389/fimmu.2023.1161145)
Supplement: Supplementary file 1 [file Table_1.docx]

Supplementary Table 1.

**Supplementary Table 1.** Summary of the different methods of inactivation and antigen preparation including inactivation agents and corresponding final concentrations, exposure time, neutralisation agents and corresponding final concentrations.

| **Inactivation method** | **[inactivation agent]** | **Exposure time** | **Neutralisation agent** | **[Neutralisation agent]** |
| --- | --- | --- | --- | --- |
| Binary ethylenimine (BEI) 0.2M | 0.10% | 1h | Sodium thiosulphate 1M | 10% |
|  | 0.10% | 4h | Sodium thiosulphate 1M | 10% |
|  | 0.10% | 6h | Sodium thiosulphate 1M | 10% |
|  | 0.04% | 4h | Sodium thiosulphate 1M | 10% |
|  | 0.04% | 6h | Sodium thiosulphate 1M | 10% |
|  | 0.04% | 9h | Sodium thiosulphate 1M | 10% |
|  | 0.04% | 18h | Sodium thiosulphate 1M | 10% |
|  | 0.04% | 22h | Sodium thiosulphate 1M | 10% |
| Formaldehyde solution (37% (w/v)) | 5% | 2h | Sodium bisulphite | 0.035% |
|  | 5% | 4h | Sodium bisulphite | 0.035% |
|  | 5% | 12 | Sodium bisulphite | 0.035% |
|  | 0.30% | 4h | Sodium bisulphite | 0.035% |
|  | 0.30% | 8h | Sodium bisulphite | 0.035% |
|  | 0.30% | 12h | Sodium bisulphite | 0.035% |
|  | 0.30% | 24h | Sodium bisulphite | 0.035% |
|  | 0.30% | 48h | Sodium bisulphite | 0.035% |
|  | 0.30% | 60h | Sodium bisulphite | 0.035% |
|  | 0.01% | 12h | Sodium bisulphite | 0.035% |
|  | 0.01% | 24h | Sodium bisulphite | 0.035% |
|  | 0.01% | 48h | Sodium bisulphite | 0.035% |
|  | 0.01% | 60h | Sodium bisulphite | 0.035% |
| Heat inactivation | Heat shock 45°C |  | 1h |  |
|  | Heat shock 50°C |  | 1h |  |
|  | Heat shock 52°C |  | 1h |  |
|  | Heat shock 54°C |  | 0.5h |  |
|  | Heat shock 56°C |  | 0.5h |  |
|  | Heat shock 60°C |  | 0.5h |  |
| Freeze-thaw cycles | (-80/-20/4/-80) x 2 |  |  |  |
|  | (-80/-20/4/-80) x 3 |  |  |  |
| Virus extract | Protein extract 1 |  |  |  |
|  | Protein extract 2 |  |  |  |
|  | DNA extract 1 |  |  |  |
|  | DNA extract 2 |  |  |  |
